# CM082 suppresses hypoxia-induced retinal neovascularization in larval zebrafish

**DOI:** 10.3389/fphar.2024.1336249

**Published:** 2024-07-29

**Authors:** Jun-long Zhang, Ding-gang Fan, Wu Yin, Bing Hu

**Affiliations:** ^1^ Division of Life Sciences and Medicine, University of Science and Technology of China, Hefei, Anhui, China; ^2^ Department of Geriatrics, Gerontology Institute of Anhui Province, The First Affiliated Hospital of USTC, Division of Life Sciences and Medicine, University of Science and Technology of China, Hefei, Anhui, China; ^3^ Anhui Province Key Laboratory of Geriatric Immunology and Nutrition Therapy, The First Affiliated Hospital of USTC, Division of Life Sciences and Medicine, University of Science and Technology of China, Hefei, Anhui, China

**Keywords:** ocular neovascular disease, CM082, retinal neovascularization, visual function, zebrafish

## Abstract

Retinal neovascularization is a common feature of several ocular neovascular diseases, which are the leading cause of blindness in the world. Current treatments are administered through invasive intravitreal injections, leading to poor patient compliance, serious ocular complications and heavy economic burdens. Thus, an alternative less or non-invasive therapeutic strategy is in demand. Here, a non-invasive oral tyrosine kinase inhibitor, CM082, was evaluated in a retinal neovascularization model induced by hypoxia in zebrafish larvae. We found that CM082 effectively suppressed retinal neovascularization, rescued cell loss in the retinal ganglion cell layer, and rescued the visual function deficiency. Our results elucidated that CM082 mediated its therapeutic efficacy primarily through the inhibition of Vegfr2 phosphorylation. The findings demonstrated that CM082 possessed strong antiangiogenic effects and may serve as a potential treatment for angiogenesis in ocular neovascular diseases.

## Introduction

Retinal neovascularization is a typical feature of several ocular neovascular diseases, including wet age-related macular degeneration (AMD), retinopathy of prematurity (ROP) and diabetic retinopathy (Lim et al., 2012; Campochiaro, 2015; Mitchell et al., 2018; Gundlach et al., 2021; Park et al., 2021). These diseases cause blood leaking, edema, retinal detachment, hence impaired visual functions and even led to blindness in patients all over the world (Rama et al., 2015; Bourne et al., 2017).

In physiological condition, angiogenesis is the normal formation of new capillaries from existing blood vessels (Pauty et al., 2018). During this process, endothelial cells (EC) proliferate, migrate and sprout to form new blood vessels from existing ones (Bielenberg and Zetter, 2015). Vascular endothelial growth factor (VEGF) with the receptor (VEGFR), platelet-derived growth factor (PDGF) with the receptor (PDGFR) and fibroblast growth factor (FGF) with the receptor (FGFR) signaling pathways play prominent roles in regulating angiogenesis (Zhao and Adjei, 2015). Among them, VEGFA and VEGFB are the decisive factors in angiogenesis, especially VEGFA, whose interaction with VEGFR2 makes the most remarkable contribution (Dvorak, 2002; Shibuya, 2013; Zhao and Adjei, 2015). After bound by VEGFA, VEGFR2 is phosphorylated, trafficked and activates various signaling molecules in EC (Lampugnani et al., 2006; Nakayama et al., 2013; Waters et al., 2021). Thus, VEGFR2 has been regarded as a novel therapeutic target for cancer and ocular neovascular diseases, where aberrant angiogenesis is prevalent (Ellis and Hicklin, 2008; Dan et al., 2021; Waters et al., 2021).

For ocular neovascular diseases, the standard therapy is monthly intravitreal injections of anti-VEGF agents, such as bevacizumab, ranibizumab, conbercept and aflibercept (Zhang et al., 2012; Cui and Lu, 2018; Mitchell et al., 2018; Apte et al., 2019). However, repeatedly long-term invasive intravitreal injections lead to poor patient compliance, various ocular complications (e.g., endophthalmitis, ocular hypertension, vitreous hemorrhage, retinal tear, and detachment) and heavy economic burdens (Thrimawithana et al., 2011; Bracha et al., 2018). There has been a calling for alternative therapeutic strategies which can be applied less invasively and more affordable to the patients.

Zebrafish (*Danio rerio*) is a well-established vertebrate model with advantages including optical transparency during larval stage, close parallelism (71%) with human disease genes, fast development and easy access (Howe et al., 2017). Moreover, zebrafish retina is similar to human in structure and function, making the larval zebrafish an ideal model for ocular neovascular diseases (Alvarez et al., 2007; Cao et al., 2008; Wu et al., 2015).

CM082 (X082, vorolanib) is a novel multitarget small molecular inhibitor which suppresses neovascularization by inhibiting VEGF, PDGF, Flt-3 and c-kit receptor tyrosine kinases (Ren et al., 2017; Xu et al., 2020). As a novel derivative of sunitinib, CM082 exhibits a more favorable toxicity and is an excellent receptor inhibitor of VEGF and PDGF in cancers (Ren et al., 2017; Dan et al., 2021). CM082 can be administered orally. This is more convenient and safer than invasive intravitreal injections (Ren et al., 2017). CM082 has been studied extensively in cancers (Zhang et al., 2020; Pedersen et al., 2021; Song et al., 2021; Bagegni et al., 2022; Liang et al., 2022), human umbilical vein endothelial cells (HUVECs) (Dan et al., 2021; Liang et al., 2022) and oxygen-induced retinopathy (OIR) models (Ren et al., 2017; Dan et al., 2021), however, little is known about the efficacy of CM082 in hypoxia-induced retinal neovascularization model in zebrafish.

To answer this question, we took advantage of an endothelial reporter line, Tg (*kdrl*:EGFP), combined with molecular methods and *in vivo* imaging. We found that CM082 exhibited a high anti-angiogenic ability possibly via *vegfr2* signaling pathway in hypoxia-induced retinal neovascularization model in zebrafish larvae. Anatomical results indicated that CM082 rescued cell loss in the retinal ganglion cell (RGC) layer. In behavioral assays, CM082 rescued the impaired optokinetic response (OKR) and visual motor response (VMR) induced by hypoxia. All in all, CM082 showed potent anti-angiogenicability, rescuing both phenotypic and functional defects in hypoxia-induced retinal neovascularization.

## Materials and methods

### Zebrafish lines and maintenance

AB/wild type (WT) and transgenic zebrafish Tg (*kdrl*:EGFP) (Beis et al., 2005) labeling endothelial cells were used in this study. During the experiment, all zebrafish embryos were raised on a 14 and 10-h light/dark cycle at 28.5°C. Zebrafish embryos were obtained from natural spawning and staged by days post-fertilization (dpf) according to established criteria (KIMMEL et al., 1995). To prevent pigment formation, larvae were maintained in embryo medium (EM) containing 0.2 mM *N*-phenylthiourea (Sigma-Aldrich, St. Louis, MO, United States). All animal manipulations in this study were performed in compliance with the guidelines and regulations set forth by the University of Science and Technology of China (USTC) Animal Resources Center and University Animal Care and Use Committee. The protocol was approved by the Committee on the Ethics of Animal Experiments of the USTC (Permit Number: USTCACUC1103013).

### Experimental hypoxia

The hypoxia device was designed to perfuse nitrogen (N_2_) gas directly into EM via an air diffuser and 1 L screw capped bottles were completely sealed to avoid air leaks (Cao et al., 2010). An oxygen electrode was fixed on the cap of the bottle to accurately detect oxygen levels when N_2_gas was directly dispersed into EM. The calibration concentration was 100% of the ambient oxygen in the air (7.80 ppm). The oxygen concentration inside the bottles were recalibrated to 20% air saturation (1.56 ppm) at each 12-h intervals. 60 mL air was injected into the bottle after every recalibration. To ensure that the EM oxygen concentration within each bottle was always consistent, we connected all bottles by air conduits. Larval zebrafish was exposed in this hypoxic condition from 1 to 5 dpf.

### CM082 treatment

CM082 was provided by Shanghai xingmo, Inc. (Shanghai, China). It was diluted with dimethyl sulfoxide (DMSO; sangon, China) and stocked at 4°C. To assess the efficacy of CM082, we placed 1 dpf larvae into EM containing either 0.5 μM or 1 μM CM082 in subsequent experiments.

### *In vivo* imaging of retinal vessels

Larvae to be imaged were transferred to EM containing 0.2 mM PTU to inhibit pigmentation at 1 dpf. Before imaging, larvae were anesthetized in MS222 (Sigma, United States) and fixed in 1% low-melting-point agarose (Sangon, China). The larvae were immediately mount on a glass-bottom imaging dish with low-melting-point agarose and imaged with an inverted confocal microscope (LSM880; Carl Zeiss, Oberkochen, Germany) under a 20× objective. The left and right were randomly selected for imaging. Approximately 40–60 3-μm optical slices were acquired every 2–4 min. Each stack was compressed to a maximal projection via Zeiss LSM software. The vessel diameter and the ratio of the area of intraocular vessel relative to the area of IRV (IV/IRV) were quantized by ImageJ software (NIH, Bethesda, MD, United States).

### Hematoxylin-eosin (HE) staining

A longitudinal tissue section (4 μm) was attached to a slide. Dried slides were removed from the oven and the paraffin was immediately put in dimethylbenzene for 10 min. Then, slides were transferred to 100% alcohol, 90% alcohol, 80% alcohol, and 70% alcohol for about 5 min each. Slides were washed in water for 2 min and moved into distilled water for 2 min. Slides were stained with hematoxylin for 10 min, and rinsed with water for 2 min. Slides were treated with 1% hydrochloric acid alcohol for 2 s, rinsed with water for 15 min and washed with distilled water for 2 s. Eosin staining last for 10 min, then tapped for each alcohol container for 5–10 min. Then slides were immersed in dimethylbenzene for 10 min. Slides were covered with coverslips, and imaged using PANNORAMIC MIDI II (3DHISTECH, Hungary). We manually counted the total number of cells in the ganglion cell layer (the yellow area of Figure 3I).

### Quantitative real-time polymerase chain reaction (qRT-PCR) and Western blots

Total mRNA was extracted by RNAiso Plus reagent (Takara) from 40 larval heads with three technical repeats. RNA was reversely transcribed into cDNA via a HiScriptII Q RT SuperMix (Vazyme, China), and analyzed by qRT-PCR using AceQ qPCR SYBR Green Master Mix (Vazyme, China) on a LightCycler 96 System (Roche Life Science, United States). All primers used in this study are given in Table 1.

**TABLE 1:** Primers used in the study.

Gene	Note	Forward primer (5′-3′)	Reverse primer (5′-3′)
*hif1α*	qRT-PCR	CCT​TCT​GCC​ACT​CAC​TCT​GTG​T	CGA​GGA​GGG​TAA​GGG​TTG​GA
*vegfr1*	qRT-PCR	CTG​CTG​AGT​GGA​ACT​CCA​GG	TGC​GTT​TGC​TGA​TAA​TGG​CG
*vegfr2*	qRT-PCR	CCA​TCG​AAC​CAG​AAA​GAC​CAA​G	ACG​ATT​GAT​CCG​CTC​CTT​ATG​A
*vegfr3*	qRT-PCR	CAG​AGC​TCA​GAG​GAC​GAT​GG	CAA​ACG​GCA​CGC​AGT​TGT​AA
β-actin	qRT-PCR	CCC​TGT​TCC​AGC​CAT​CCT​T	TTG​AAA​GTG​GTC​TCG​TGG​ATA​CC

To verify the protein expression of Hif1α in zebrafish larvae, the heads of 40 larvae (5 dpf) were lysed with RIPA buffer (Sangon, China). Identical quantities of pooled normoxic and hypoxic extracts were added into SDS-PAGE gels. Hif1α (1:500; NB100-134; Novus), p-Vegfr2 (pY1054/Py1063) (1:1,000; PA5881; Abmart; China), Gapdh (1:2,000; AB_3069651; Huabio; China) and β-actin (1:1,000; GTX54233; Genetex, Irvine, CA, United States) were used as primary antibodies in western blots. The HRP-conjugated goat anti-rabbit secondary antibody (Invitrogen, Carlsbad, CA, United States) was finally diluted to 1:10,000 at room temperature.

### Optokinetic response assays

Optokinetic response (OKR) assays were based on a published design (Huang et al., 2018). A sine-wave grating was generated using the computer software LabVIEW and projected by an LCD projector. Zebrafish larvae were immobilized dorsal side up in a 35-mm petri dish with 6% methylcellulose above a small infrared light. When the rotating grating patterns were projected around the larva, the evoked eye movement was recorded in real time by an infrared-sensitive CCD camera (TCA-1.3BW; Nanjing, China). The larvae of every group were stimulated with a constant angular velocity of 7.5 degree/s and a fixed spatial frequency of 0.04 cycle/degree. To measure visual acuity, the spatial frequency was presented with 0.04 and 0.06 cycle/degree. We measured the contrast sensitivity using the gain (ratio of eye velocity and stimulus velocity) of OKR (Rinner et al., 2005).

### Visual motor response assays

Visual motor response (VMR) assays were conducted as previously described (Emran et al., 2007; Emran et al., 2008). The assay was performed inside a ZebraBox system (ViewPoint Life Sciences, Lyon, France). Zebrafish larvae from 8 groups were each placed in a 48-well plate. To acclimatize the animals, the 48-well plate with zebrafish larvae was dark-adapted in the ZebraBox system for 3.5 h before the actual experiment. The light switch (on or off) was abrupt and instantaneous. The movement of larvae was summarized as the fraction of frames in which a larva displayed movement in each second. We used custom PERL software and Visual Basic Macros for Microsoft Excel to process and analyze the data.

### Statistical analyses

Statistical analyses were performed with GraphPad Prism8 software (San Diego, CA, United States). All data were presented as mean ± SEM. Statistical significance between groups was identified by Student’s *t-*test, non-parametric tests, or one-way ANOVA. Significance was classed as follows: ∗*p <* 0.05, ∗∗*p <* 0.01, and ∗∗∗*p <* 0.001. In particular, we analyzed three independent samples of three times each for the gene expression experiment to gain reliable results.

## Results

### Creation of hypoxic conditions for zebrafish larvae

**FIGURE 1:**
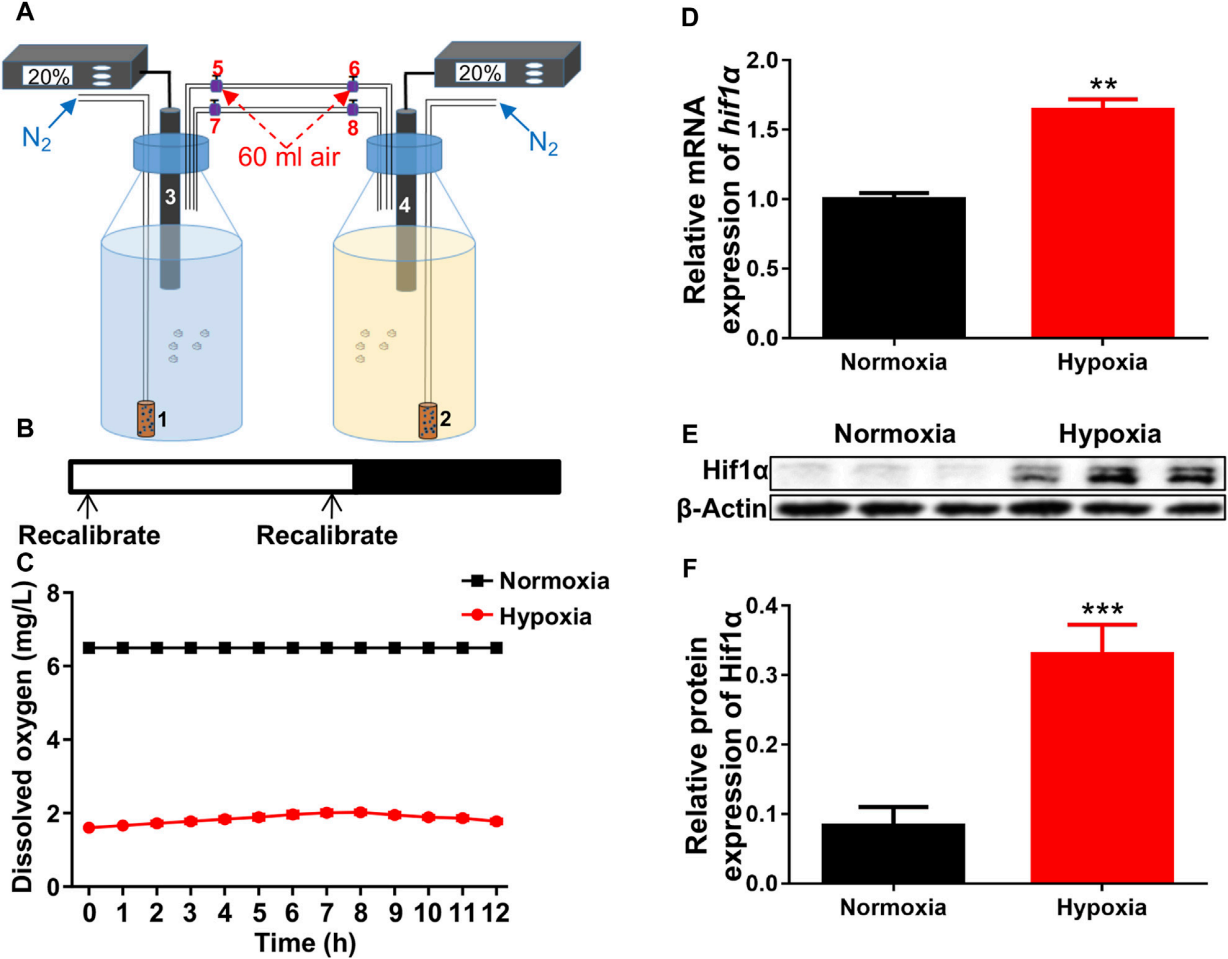
Hypoxic setup for larval zebrafish. **(A)** Pattern of hypoxic devices. 1 and 2, air diffuser; 3 and 4, oxygen electrode; 5–8, conduit connector. **(B, C)** The oxygen concentration inside the bottles were recalibrated to 20% air saturation (1.56 ppm, 1.6 mg/L) at each 12-h intervals (shown in B is not 12 h interval) from 1 to 5 dpf. **(D)** qRT-PCR analysis showed that the expression of *hif1α* was significantly increased after exposed to hypoxia. **(E, F)** Western blots showed that Hif1α protein expression was significantly increased under hypoxia. Hif1α, 110 kD; β-actin, 42 kD. *p < 0.05; **p < 0.01; ***p < 0.001.

The hypoxia was constructed as described in the methods (Figure 1A). The oxygen concentration was recalibrated to 20% air saturation (1.56 ppm) every 12 h (Figure 1B). Dissolved oxygen levels were exactly measured by the oxygen electrode once an hour. Based on preliminary research, a dissolved oxygen level of 1.6 mg/L was appropriate for our study (Figure 1C). After exposed to hypoxia, both the mRNA and protein levels of *hif1α*, the master regulator of hypoxia response, were significantly increased compared to control (Figures 1D–F), showing that we successfully created suitable hypoxic conditions for zebrafish larvae.

### CM082 suppressed hypoxia-induced retinal neovascularization

**FIGURE 2:**
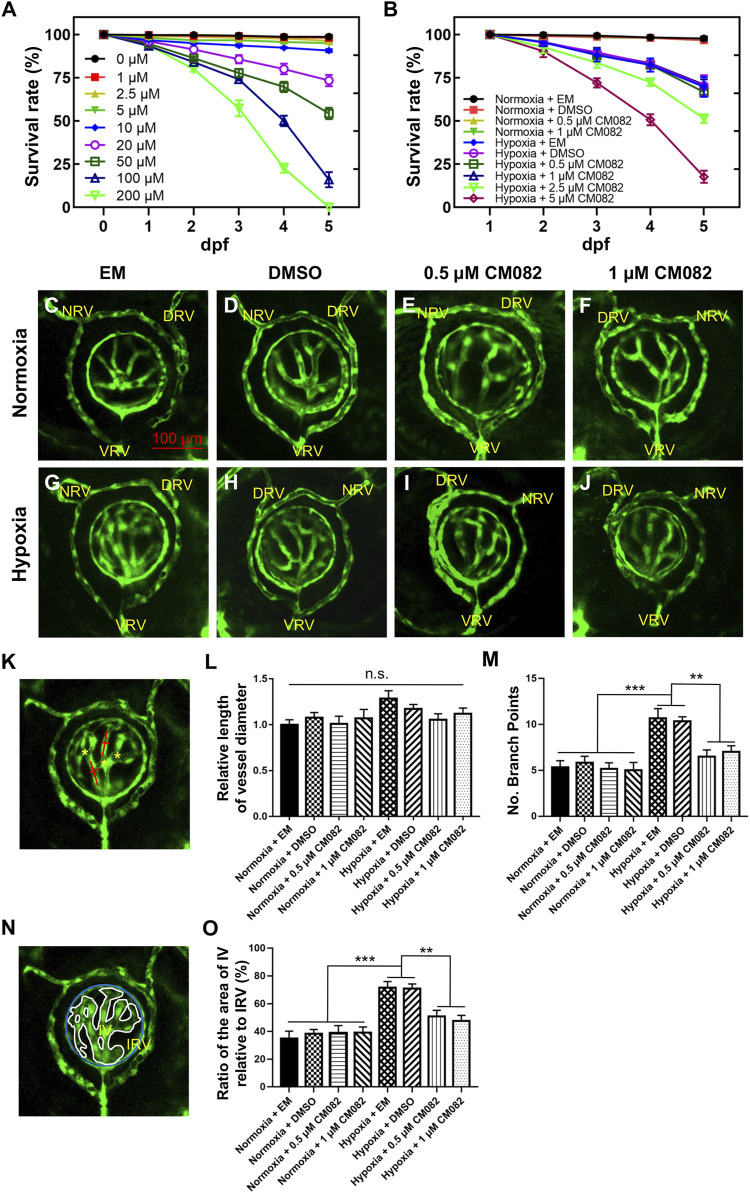
CM082 suppressed retinal neovascularization induced by hypoxia. **(A)** Survival rate of zebrafish embryos exposed to CM082 from 0 to 200 μM under normoxia (n = 100 in each group). **(B)** Survival rate of zebrafish embryos in various media under normoxia or hypoxia (n = 100 in each group). **(C–J)** The retinal vessels in various media under normoxia and hypoxia conditions were imaged (n = 6 in each group). **(K, N)** The pattern shows the method for measuring and counting vessel diameters (red line), ratio of the area of IV relative to IRV (IV, white line; IRV, blue line) and branch points (yellow asterisks). The vessel diameter and the IV/IRV ratio were measured using Image J. **(L)** There was no significant difference on the vessel diameter among all the 8 groups. **(M, O)** Under normoxia, the number of branch points and the IV/IRV ratio were not significantly different among the 4 groups with various media. However, these two values were significantly higher in the hypoxia groups with EM or DMSO compared to the normoxia groups. Under hypoxia, this discrepancy was mitigated by 0.5 and 1 μM CM082. NRV, nasal radial vessel; DRV, dorsal radial vessel; VRV, ventral radial vessel; IV, intraocular vessel; IRV, intraocular ring vessel. Scale bar = 100 μm *p < 0.05; **p < 0.01; ***p < 0.001. Data represent the mean ± SEM.

Consistent with previous studies (Wu et al., 2015), hypoxia significantly promoted angiogenesis in zebrafish retina. To identify proper concentrations of CM082 to be used in this study, we did survival test with a series of concentrations. Under normoxia, CM082 showed low toxicity when the concentration was below 10 μM (Figure 2A). However, under hypoxia, CM082 already showed high toxicity at 2.5 μM. Hence we selected to use 0.5 and 1 μM CM082 (Figure 2B) in the following experiments.

From 1 dpf onward, Tg (*kdrl*:EGFP) embryos were exposed to EM, DMSO, 0.5 or 1 μM CM082 in both normoxia and hypoxia conditions. At 5 dpf, the larvae were collected for *in vivo* imaging. To ensure the consistency, each image captured the nasal, dorsal and ventral radial vessels (NRV, DRV and VRV), and the surface and intraocular ring vessels (SRV and IRV) (Kitambi et al., 2009; Kaufman et al., 2015) (Figures 2C–J). The quantitative analysis of retinal vasculature was performed as previously described (Wu et al., 2015). Specifically, three metrics were used–vessel diameter, number of branch points and the ratio of the area of intraocular vessel relative to the area of IRV (IV/IRV) (Figures 2K, N).

Results showed that there was no significant difference on the vessel diameter among all the 8 groups (Figure 2L). Under normoxia, the number of branch points and the IV/IRV ratio were not significantly different among the 4 groups with various media (Figures 2C–F, M, O). However, these two values were significantly higher in the hypoxia groups with EM or DMSO compared to the normoxia groups (Figures 2C, D, G, H, M, O), indicating hypoxia induced retinal vascularization in zebrafish larvae. Under hypoxia, this discrepancy was mitigated by 0.5 and 1 μM CM082 (Figure 2I, J, M, O). Taken together, our results indicated that CM082 suppressed retinal neovascularization induced by hypoxia.

### CM082 rescued the number of cells in the RGC layer under hypoxia

**FIGURE 3:**
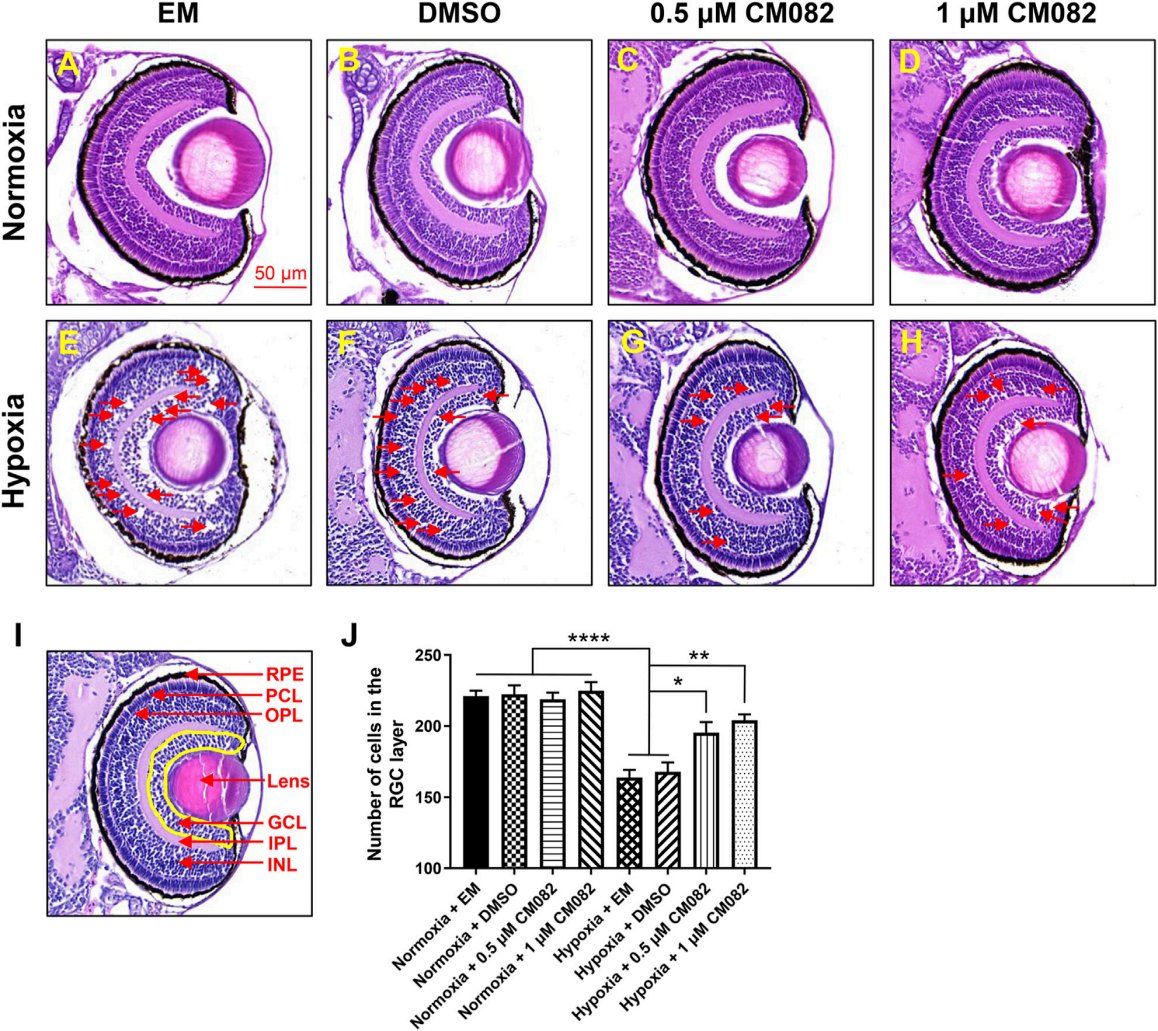
CM082 rescued cell loss in the area of GCL induced by hypoxia. **(A–H)** The eyes’ HE-staining of larval zebrafish exposed to various media in both normoxia and hypoxia conditions. There were nearly no retinal lesions under normoxia **(A–D)**. However, multiple retinal lesions appeared in the INL and GCL in hypoxia groups with EM **(E)** and DMSO **(F)** and reduced by 0.5 and 1 μM CM082 **(G, H)** (red arrowheads). **(I, J)** Quantification of cell numbers in the GCL area (yellow area) in eight groups (n = 4 in each group). Under normoxia, the cell numbers in GCL were not significantly different among the 4 groups with various media. However, there were significantly fewer GCL cells in the hypoxia groups with EM or DMSO compared to the normoxia groups, indicating hypoxia reduced the number of cells in GCL. The cell loss was partially abrogated by 0.5 and 1 μM CM082 under hypoxia. PRE, retinal pigmented epithelium; PCL, photoreceptor layer; OPL, outer plexiform layer; GCL, ganglion cell layer; IPL, inner plexiform layer; INL, inner nuclear layer. Scale bar = 50 μm *p < 0.05; **p < 0.01; ***p < 0.001. Data represent the mean ± SEM.

It has been reported that retinal neovascularization could cause breakage of the inner blood-retinal barrier, leading to fluid leakage and macular edema (Tranos et al., 2004). To determine whether hypoxia can cause macular edema, we performed HE-staining. There were nearly no retinal lesions under normoxia (Figures 3A–D). However, consistent with previous study (van Rooijen et al., 2010), multiple retinal lesions appeared in the inner nuclear layer (INL) and ganglion cell layer (GCL) in hypoxia groups with EM and DMSO (red arrowheads, Figures 3E, F), suggestive of macular edema. The edema was reduced by CM082 (red arrowheads, Figures 3G, H).

Previous study showed that retinal degenerative diseases mainly affect the outer layers of the retina, such as RPE and PCL, but there is increasing evidence that they also affect inner layers, such as GCL (Zarbin, 2016; Xuan et al., 2021). This is further verified by quantification of cells in the area of GCL (Figures 3I, J). Under normoxia, the cell numbers in GCL were not significantly different among the 4 groups with various media (Figures 3A–D, I, J). However, there were significantly fewer GCL cells in the hypoxia groups with EM or DMSO compared to the normoxia groups (Figures 3A–F, I, J), indicating hypoxia reduced the number of cells in GCL. This reduction of cell number was partially abrogated by 0.5 and 1 μM CM082 under hypoxia (Figures 3E–J). The results showed that CM082 reduced macular edema and rescued cell loss in GCL induced by hypoxia in the retina of zebrafish larvae.

### CM082 inhibited the expression of *vegfr2* in the eyes of zebrafish larvae

**FIGURE 4:**
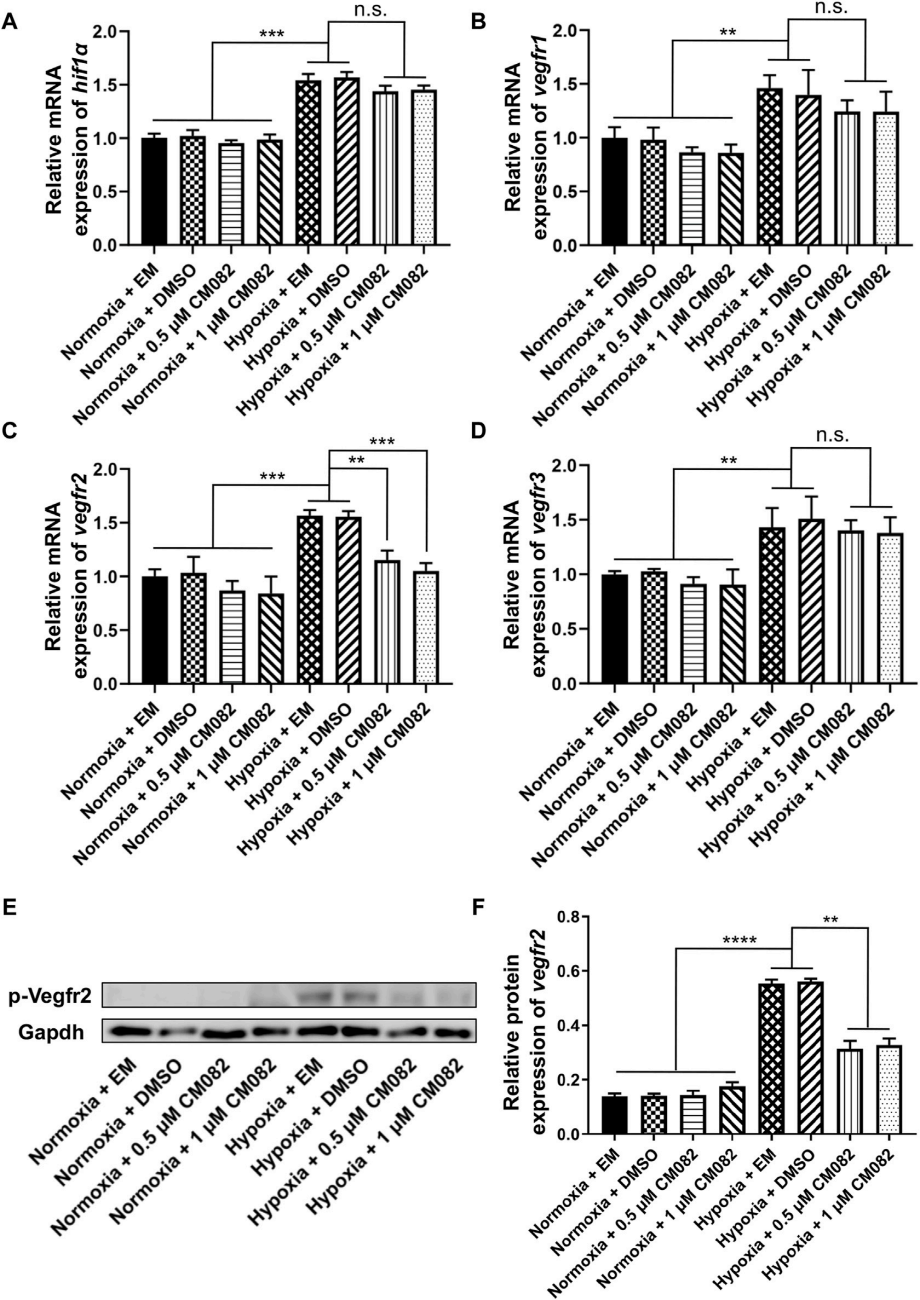
CM082 inhibited the expression of *vegfr2* in the eyes of zebrafish larvae under hypoxia. **(A–D)** qRT-PCR analysis of *hif1α*, *vegfr1*, *vegfr2* and *vegfr3* in eight groups. **(A, B, D)** The results showed higher *hif1α*, *vegfr1* and *vegfr3* mRNA levels in hypoxia than normoxia. CM082 did not further altered the mRNA level. **(C)** The expression of *vegfr2* was significantly upregulated under hypoxia, and downregulated by CM082. **(E)** Western blots of p-Vegfr2 and Gapdh. p-Vegfr2, 220 kD; Gapdh, 36 kD. **(F)** The expression of p-Vegfr2 was significantly upregulated under hypoxia, then downregulated by CM082. *p < 0.05; **p < 0.01; ***p < 0.001. Data represent the mean ± SEM.

Since *hif1α* is the main regulator during hypoxia response, we wondered if it was influenced by CM082 in hypoxia. Quantitative RT-PCR results showed higher *hif1α* mRNA levels in hypoxia than normoxia, however, CM082 did not further altered its expression (Figure 4A). Thus, the effects of CM082 were possibly mediated by other signaling molecules.

It has been reported that *vegfr1*, *vegfr2* and *vegfr3* are the targets of CM082 (Ren et al., 2017). In our assay, the same expression pattern as *hif1α* was observed in *vegfr1&3*,i.e., higher in hypoxia than normoxia, lower in hypoxia but no significant difference with CM082 in hypoxia (Figures 4B, D). However, the expression of *vegfr2* was significantly upregulated under hypoxia, then downregulated by CM082 (Figure 4C). The protein expression of p-Vegfr2 was significantly upregulated under hypoxia, then downregulated by CM082 (Figures 4E, F). These results suggest that CM082 can suppress hypoxia-induced retinal neovascularization via Vegfr2 signaling.

### CM082 rescued the visual function deficiency induced by hypoxia

**FIGURE 5:**
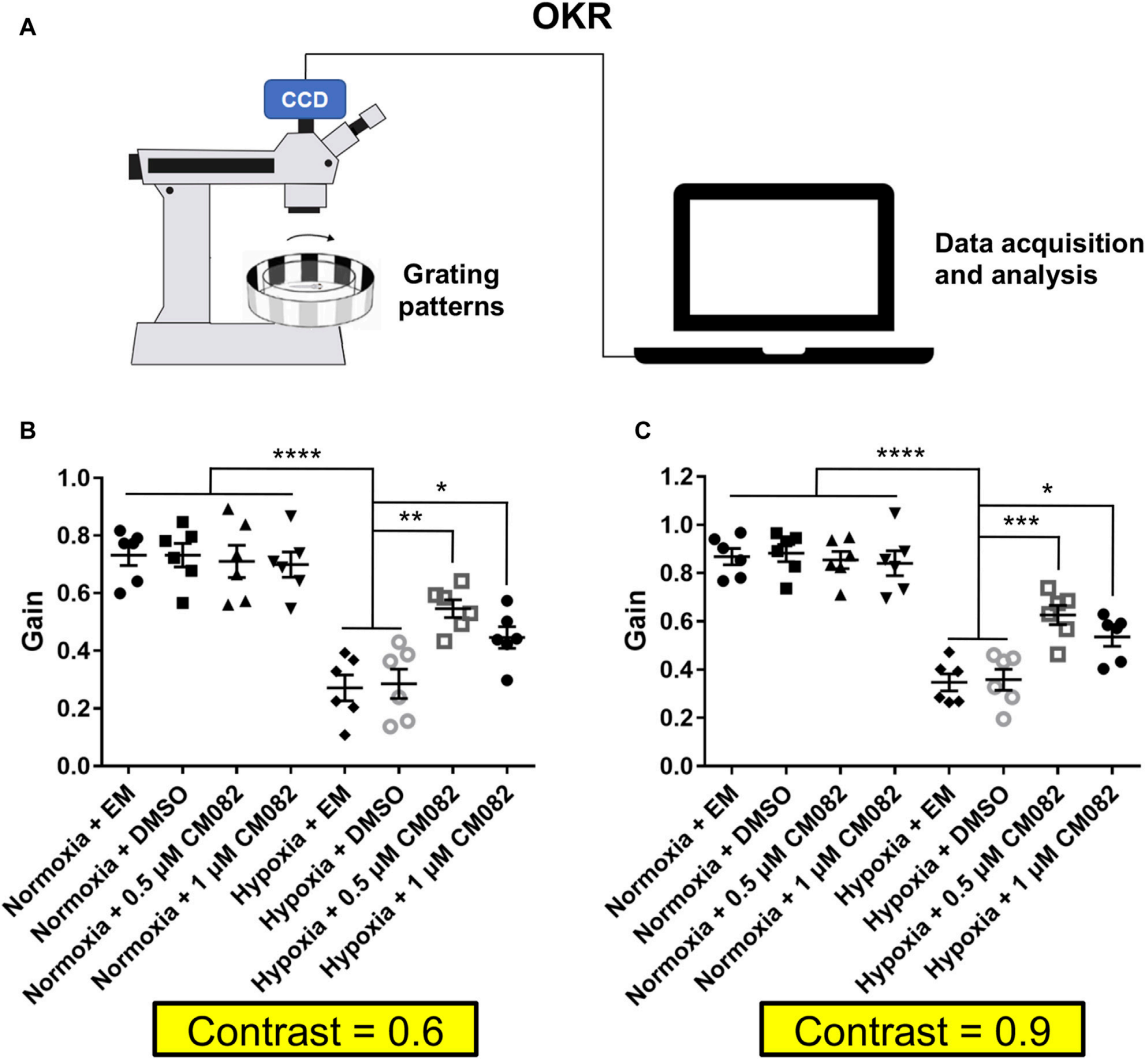
CM082 rescued optokinetic response deficiency induced by hypoxia. **(A)** Scheme of OKR. Parameters of the grating patterns, including contrast, rotating direction and velocity were controlled by the computer. The eyes of the fish larva will follow the motion of the grating patters. Movement of the eyes were recorded by the camera and analyzed by the computer. **(B, C)** OKRs of larval zebrafish which were exposed to EM, DMSO, 0.5 or 1 μM CM082 in both normoxia and hypoxia conditions (Contrast = 0.6 and = 0.9; n = 6 in each group). The gains were lower in hypoxia than normoxia both in Contrast = 0.6 and in Contrast = 0.9. CM082 treatment partially rescued the gain in hypoxic larvae. *p < 0.05; **p < 0.01; ***p < 0.001. Data represent the mean ± SEM.

Since CM082 partially alleviated retinal neovascularization induced by hypoxia, we explored if it could rescue the vision function as well. First, we collected and analyzed the contrast sensitivity with the optokinetic response (OKR) (Figure 5A). In this behavioral assay, the gain value indicates to what extent the zebrafish eyes can follow the rotation of the surrounding gratings. Higher gain means better vision function. As expected, the gain was lower in hypoxia than normoxia (Figures 5B, C), indicating deficient contrast sensitivity in hypoxia. This result was consistent with a previous study in our lab (Yang et al., 2018). CM082 treatment partially rescued the gain in hypoxic larvae, although not to the control level (Figures 5B, C).

**FIGURE 6:**
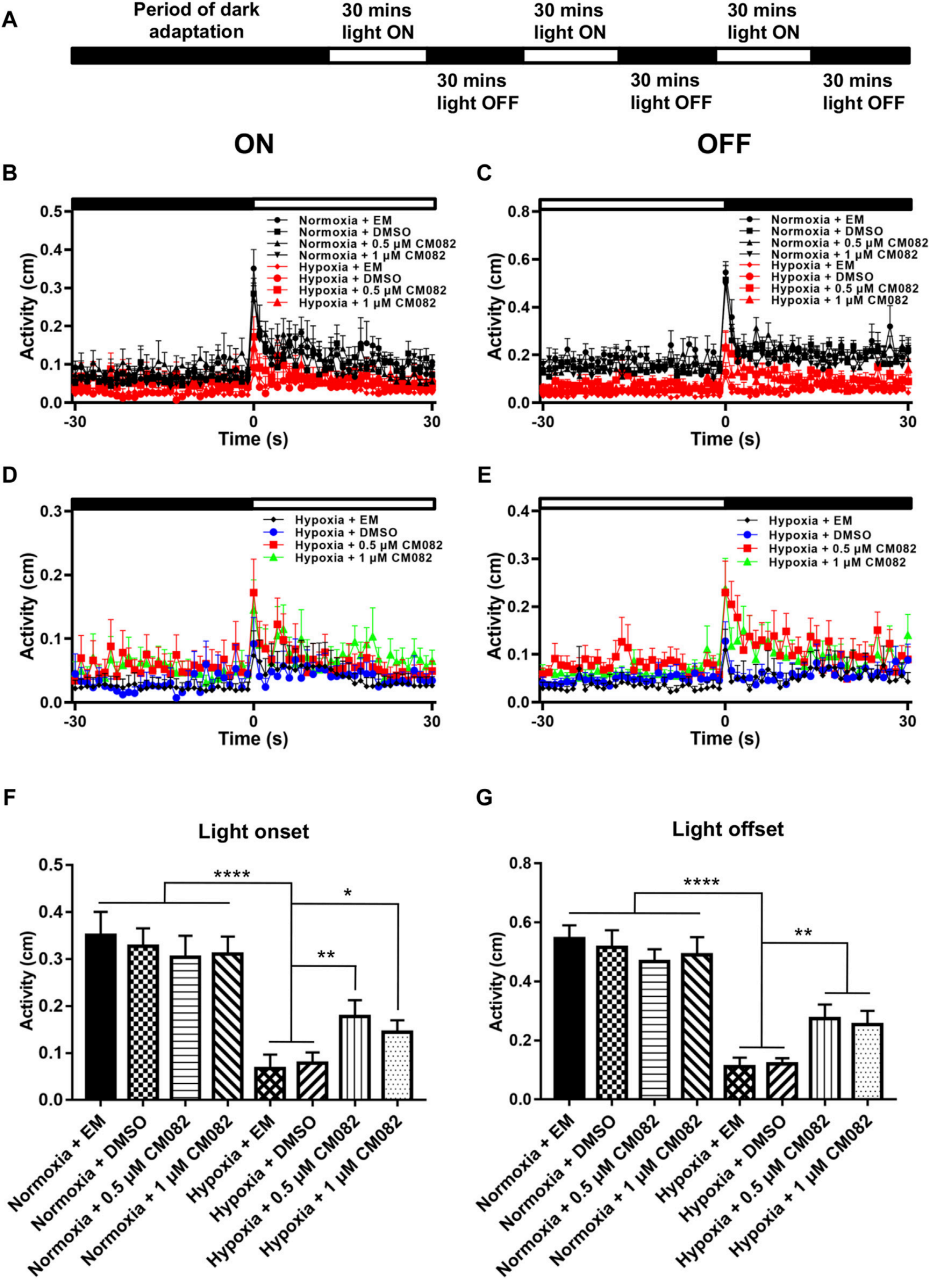
CM082 partially rescued visual motor response (VMR) deficiency induced by hypoxia. **(A)** Time frame of VMR. **(B, C)** Motor activity in response to light-on and light-off in larval zebrafish which were exposed to EM, DMSO, 0.5 or 1 μM CM082 in both normoxia and hypoxia conditions. The activities of normoxia groups are higher than hypoxia groups. **(D, E)** Motor activity in response to light-on and light-off in larval zebrafish which were exposed to EM, DMSO, 0.5 or 1 μM CM082 under hypoxia. **(F, G)** The activity of light onset and light offset in eight groups. There was no significant difference among the normoxic groups. However, the activity in hypoxia was significantly reduced and it was partially rescued by CM082. n = 12 in each group. *p < 0.05; **p < 0.01; ***p < 0.001. Data represent the mean ± SEM.

Next, we analyzed the primitive startle response. It is an unconditioned reaction stimulated by the sudden change in ambient light (Emran et al., 2007; Zhang et al., 2016). The VMR experiments were performed as described in Figure 6A. Similar to the OKR results, larvae in hypoxia responded weakly at light onset and offset compared to normoxia (Figures 6B, C). To clearly visualize the differences among the hypoxic groups, the VMR results of hypoxia were displayed in individual figures (Figures 6D, E). The distances of moving in 1 s right after light-on and off were increased by CM082 (Figures 6D, E), indicating an improvement in vision function.

Moreover, we quantified the distance in 1 s at light-on and off (Figures 6F, G). There was no significant difference among the normoxic groups. However, the distance in hypoxia was significantly reduced, and once again, was partially rescued by CM082 (Figures 6F, G). In one word, CM082 could rescue the visual function deficiency induced by hypoxia.

## Discussion

AMD is a retinal disease that causes visual impairment and severe vision loss (Mitchell et al., 2018). Zebrafish models are becoming popular in studying retinopathy (Cao et al., 2008; Wu et al., 2015). In this study, we exposed Tg (*kdrl*:EGFP) zebrafish larvae to hypoxia to model the AMD and retinal neovascularization. Expression of *hif1α* was increased at both mRNA and protein levels in response to hypoxia. Moreover, retinal neovascularization was induced by hypoxia in the eyes of zebrafish larvae, consistent with previous works (Cao et al., 2008; Cao et al., 2010; Wu et al., 2015). A recent study showed that CM082 can significantly inhibit VEGF-mediated cell proliferation, migration, invasion, and tube formation (Dan et al., 2021). Another study demonstrated that oral administration of CM082 can pass through the blood-retinal barrier and significantly inhibit laser-induced choroidal neovascularization in rats (Ren et al., 2017). Our results showed that CM082 significantly suppressed retinal neovascularization and macular edema and significantly rescued cell loss in the area of GCL induced by hypoxia in zebrafish larvae. These results have not been mentioned in previous reports.

Moreover, our results demonstrated that CM082 significantly inhibited the gene expression of *vegfr2* in the eyes of zebrafish larvae under hypoxia. However, there was little change in the gene expression of *vegfr1* and*vegfr3* when CM082 was applied. These results indicated that CM082 can exerted the rescuing effects by inhibiting the expression of p-Vegfr2 in zebrafish larvae.

Furthermore, larval zebrafish under hypoxia showed deficient visual functions as revealed by OKR and VMR assays and were rescued by CM082. In a phase I clinical trial to evaluate the preliminary efficacy of CM082, the results indicated that participants required lower anti-VEGF injections than expected (Jackson et al., 2017). Current treatments for ocular neovascular diseases are administered through invasive intravitreal injections and need a cold chain storage. On contrary, CM082 can be taken orally and is safer, less invasive and more convenient.

In summary, CM082 effectively suppressed hypoxia-induced retinal neovascularization and macular edema and rescued cell loss in the area of GCL induced by hypoxia possibly by inhibiting the gene expression of *vegfr2* in zebrafish larvae. It also rescued the visual function deficiency induced by hypoxia. Therefore, CM082 may be a potential treatment for angiogenesis in ocular neovascular diseases in the future.

## Data Availability

The original contributions presented in the study are included in the article/Supplementary Material, further inquiries can be directed to the corresponding authors.
